# Oral microbiome and postmenopausal breast cancer risk: findings from the NIH-AARP diet and health study

**DOI:** 10.1080/20002297.2026.2728745

**Published:** 2026-10-09

**Authors:** Giovanny Herrera, Xing Hua, Zeni Wu, Linda M. Liao, Yunhu Wan, Casey L. Dagnall, Kristine Jones, Belynda D. Hicks, Amy Hutchinson, Mitchell H. Gail, Jianxin Shi, Gretchen L. Gierach, Rashmi Sinha, Emily Vogtmann

**Affiliations:** a Division of Cancer Epidemiology & Genetics, National Institutes of Health, Bethesda, MD, USA; b Frederick National Laboratory for Cancer Research/Leidos Biomedical Research Laboratory, Inc., Frederick, MD, USA; c School of Population Medicine and Public Health, Chinese Academy of Medical Sciences & Peking Union Medical College, Beijing, China

**Keywords:** Oral microbiome, breast cancer risk, prospective cohort study, microbial diversity, cancer epidemiology

## Abstract

**Introduction:**

Breast cancer is the most frequently diagnosed cancer among women worldwide. Some case-control studies have reported associations between the oral microbiome and breast cancer, but the temporality of the association remains unclear. Therefore, we examined this association prospectively in the NIH-AARP Diet and Health Study.

**Methods:**

In 2005, oral wash samples were collected from a subset of the NIH-AARP cohort. We identified 318 women who developed breast cancer after collection and 318 controls, incidence-density matched on age at collection, race/ethnicity and smoking. DNA was extracted using the DNeasy PowerSoil Pro Kit, followed by shotgun metagenomic sequencing. Conditional logistic regression was used to calculate the odds ratios (OR) for associations between alpha- and beta-diversity and species- and gene-level metrics with breast cancer risk, adjusting for potential confounders.

**Results:**

Cases developed breast cancer on average 3.4 years after sample collection. Overall, the majority of the microbial metrics, including species and genes, showed largely null associations with the exception of Pielou's evenness (OR = 0.82; 95% CI = 0.70–0.95). When stratified by time to diagnosis, the inverse association with Pielou's evenness was only detected among women diagnosed within 2 years (29% of cases). In this subgroup, some previously reported associations, like the inverse association with the presence of *Treponema* (OR = 0.37; 95% CI = 0.17–0.78), were replicated. Null associations were predominantly observed for cases diagnosed more than 2 years after sample collection.

**Conclusions:**

The oral microbiome was generally not prospectively associated with postmenopausal breast cancer in a US population and results suggested previous findings may reflect reverse causation.

## Introduction

Breast cancer is the most frequently diagnosed cancer in women and the second leading cause of cancer-related death after lung cancer, with an estimated 2.3 million new cases and 670,000 deaths globally in 2022 [1]. Several established risk factors influence breast cancer development, including age at menarche, age at first birth, age at menopause, parity, alcohol use, smoking and obesity [1,2]. Additional environmental, biological and genetic factors have also been described; however, over 50% of women diagnosed with breast cancer do not have identifiable risk factors or a genetic predisposition [3,4], suggesting that other contributing factors need to be explored.

The oral microbiome is a complex ecosystem that has been linked to various health conditions [5–8]. For instance, certain oral microorganisms can drive inflammatory diseases such as periodontitis, wherein pathogenic bacteria, particularly those from the red and orange complexes, proliferate and trigger destructive host immune responses [9–12]. Similarly, alterations in the oral microbiome, especially those linked to periodontal disease, have been hypothesized to contribute to breast cancer risk, although findings across studies have been inconsistent [13–19].

We previously conducted a case-control study of the oral microbiome and breast cancer in 881 women in Ghana (369 breast cancer cases, 93 non-malignant breast disease cases and 419 population based controls), which is the largest study of its kind to date, and detected inverse associations between specific periodontal pathogens, particularly those belonging to the red and orange complexes, as well as associations with other oral bacterial genera with breast cancer case status [16]. However, given the cross-sectional nature of this study, it was unclear whether the oral microbiome differences preceded breast cancer development. In contrast, a study conducted in Canada analyzed saliva samples from 54 breast cancer cases and 218 controls that had been retrospectively collected a median of six years after the diagnosis, as well as prospectively collected samples from 82 breast cancer cases and 82 controls with a median of four years before diagnosis. This study reported limited associations between the oral microbiome and breast cancer in the cross-sectional analysis and found no significant associations in the prospective setting [19].

To better understand the potential involvement of the oral microbiome in the etiology of breast cancer, we conducted a prospective analysis of the association between the oral microbiome and breast cancer using prediagnostic oral samples from women in the NIH-AARP Diet and Health study.

## Materials and methods

### Study population

Between 1995 and 1996, AARP members aged 50–71 residing in six U.S. states (California, Florida, Louisiana, New Jersey, North Carolina and Pennsylvania) or two metropolitan areas (Atlanta, GA and Detroit, MI) were invited to participate in the NIH-AARP Diet and Health Study, resulting in a baseline cohort of 566,398 individuals [20]. Dietary and lifestyle data were collected via a standardized questionnaire. Baseline dietary intake was assessed using a 124-item food-frequency questionnaire (FFQ), an early version of the National Cancer Institute Diet History Questionnaire (DHQ), calibrated against two non-consecutive 24-h dietary recalls in a sub-study of 1,953 individuals [21,22]. In 2005, 34,262 oral wash specimens (collected using Scope mouthwash, Procter & Gamble) were mailed to a subset of participants including participants who had previously developed colorectal, breast, or prostate cancer. Incident breast cancer diagnoses were identified through 31 December 2011, via linkage to state cancer registries and mortality information was available through linkage to the National Death Index. Among women without a prior breast cancer diagnosis at the time of oral wash collection in 2005, 348 incident breast cancer cases between 59.8 and 79.8 years of age (at sample collection) were identified during follow-up. Each case was matched to a control using incidence density sampling, with matching criteria including age at sample collection, self-reported race/ethnicity and baseline smoking history (current, former, never). One matched control was included twice because she later developed breast cancer and was included as a case and a matched control. Based on age at sample collection, the included breast cancer cases were likely postmenopausal; however, menopausal status was not reassessed at that time.

### DNA extraction and sequencing

The pre-treatment of oral wash samples was described previously [23]. In brief, upon receipt, samples were centrifuged at 4,000 × *g* for 5 min, followed by an additional centrifugation at 7,000 × *g* until a visible pellet was formed. The supernatant was discarded, and the cell pellets were resuspended in 1× Tris-EDTA buffer, aliquoted into sterile cryovials and stored at −70°C until DNA extraction. A total of 15 DNA extraction batches were created with matched case-control pairs included in the same batch along with 6 quality control samples: two bacterial artificial communities (the Microbiome Quality Control Project [MBQC] oral artificial community [24] and the ZymoBiomics Microbial Community Standard [Zymo Research, Irvine, CA]), a mouthwash from a generous donor, two extraction replicates and one extraction blank (TE buffer). DNA extractions were performed at the Cancer Genomics Research Laboratory (CGR) at the National Cancer Institute (NCI) in Rockville, Maryland, using the DNeasy PowerSoil Pro kit on the QIAcube HT automated purification system (Qiagen, Hilden, Germany). DNA was quantified using the Quant-iT PicoGreen dsDNA Assay Kit (Thermofisher, Waltham, MA). DNA extraction failed for ten samples, including eight controls and two cases (Figure S1).

Briefly, 50 ng of genomic DNA was purified using the Agencourt AMPure XP Reagent (Beckman Coulter Life Sciences, Indianapolis, IN) following the manufacturer's instructions. Library preparation was performed using the Roche KAPA HyperPlus Kit (Roche, Indianapolis, IN) with xGen Dual Index UMI Adapters (IDT, Coralville, IA), as per Roche's recommended protocol. Fragmentation conditions were optimized to yield an average insert size of 350 bp. Library amplification was carried out via ligation-mediated PCR using a reaction mixture containing 20 μL of library DNA, 25 μL of 2 × KAPA HiFi HotStart ReadyMix and 5 μL of 10 × Library Amplification Primer Mix (primers: 5′-AATGATACGGCGACCACCGA-3′ and 5′-CAAGCAGAAGACGGCATACGA-3′). The PCR cycling conditions included an initial denaturation at 98°C for 45 s, followed by 7 cycles of 98°C for 15 s, 60°C for 30 s and 72°C for 30 s, with a final extension at 72°C for 1 min. Post-amplification, libraries were purified using two consecutive 1 × bead cleanups with Agencourt AMPure XP Reagent, according to the Roche protocol. Quantification of the amplified libraries was performed with Roche's Library Quantification Kit for Illumina on a LightCycler 480 system (Roche). The multiplexed libraries were then sequenced on an Illumina NovaSeq X platform (San Diego, CA) using 2 × 150 bp paired-end reads, achieving a mean read depth of 51 million reads per sample.

### Bioinformatic analysis

The raw sequencing reads from shotgun metagenomic sequencing were quality trimmed and filtered using BBDuk from the BBTools suite [25]. Host DNA was subsequently removed by aligning the reads to the human reference genome CHM13 v2.0 [26]using Bowtie2 [27]. Taxonomic profiling of the remaining unmapped reads was performed with MetaPhlAn4 [28], and functional profiling was conducted using HUMAnN3 [29] and the UniRef90 database [30]. This process identified 767 unique species, 841,987 genes and 514 pathways across samples.

Following rarefaction to 1 million reads per sample (Figure S2A), 24 samples were dropped. Participants with excluded samples did not differ from those retained in the main analysis with respect to any of the variables examined (data not shown). Additionally, 26 samples that lacked matched pairs (17 cases and 9 controls) were excluded from the downstream analysis (Figure S1). We computed alpha diversity (observed species, Shannon index, Pielou's evenness index) and beta diversity (Bray–Curtis and Jaccard distances) metrics for both study samples (*n* = 635) and replicates (*n* = 15) from the taxonomic tables. Principal coordinate analysis (PCoA) vectors were calculated for the beta diversity matrices. The relative abundances and centered log ratio (CLR) transformed relative abundances at the species-level were calculated. For presence/absence analyses, species detected in fewer than 5% or more than 95% of samples were excluded. For relative abundance analyses, species with a mean relative abundance below 0.1% were excluded. Functional analyses included exclusively the reads that could be assigned to known genes or pathways. Copies per million (for genes) and relative abundances (for pathways) were calculated. Genes with fewer than 100 copies per million and pathways with a relative abundance below 0.1% were excluded. For presence/absence analysis, we only included genes and pathways with a prevalence between 5 and 95%.

### Quality control

Differences between excluded women (due to failed DNA extraction or low read counts) and included participants were evaluated using chi-square, Fisher's exact, or Wilcoxon tests, and no significant differences were found. PCoA plots generated from Bray–Curtis and Jaccard distance matrices showed a distinct cluster of the artificial communities, which were separated from the other samples included (study samples, replicates and generous donor samples) (Figure S2B). For the positive controls, the median coefficient of variation (CV) for the alpha diversity metrics was 9.15% in the ZymoBiomics communities, 6.18% for the MBQC artificial community and 1.22% for the generous donor mouthwash; the median CV for all species with a relative abundance > 0.01% was 14.3% for the generous donor mouthwash, suggesting high inter-batch reliability. Median intraclass correlation coefficients (ICCs) for alpha diversity and species (relative abundance > 0.01%) based on the extraction replicates were 0.98 and 0.99, respectively, suggesting high intra-batch reliability. For the negative control samples, the median number of reads was 51,134 (ranging from 0 to 21 million reads). After rarefaction, the majority of the blanks were removed, but two blanks out of 15 remained with 3 and 21 million reads. The blank with 3 million reads contained only one taxon, *Sphingomonas echinoides*, which has previously been described in the oral microbiome [31], so this taxon was retained in downstream analyses. In contrast, the blank with 21 million reads exhibited a taxonomic profile similar to the study samples, likely indicating some cross-contamination from a nearby sample during DNA extraction. It is worth noting that all results were similar after excluding the two batches with potential cross-contamination.

### Statistical power for analysis

We calculated the minimum OR( $=e^{\beta }$ ) for detecting an association between the relative abundance of a specific taxa with breast cancer risk with 80% statistical power, based on the *p*-value cutoffs α= 0.05, 0.01 and 0.001 using the formula $\beta ={(C}_{1-\frac{\alpha }{2}}+C_{0.8})/\sqrt{\frac{1}{n_{1}}+\frac{1}{n_{2}}}$ with $n_{1}=n_{2}=318$ representing the number of cases and matched controls. For the *p*-value cutoffs of α= 0.05, 0.01 and 0.001, the minimum detectable ORs were 1.25, 1.31 and 1.39, respectively.

### Statistical analysis

Characteristics of study participants were summarized by breast cancer status. Differences in alpha diversity indices between cases and controls were evaluated using the Wilcoxon signed-rank test. Conditional logistic regression models were used to calculate odds ratios (OR) and 95% confidence intervals (CI) for the association between the oral microbiome (alpha diversity, species presence or relative abundance [with and without CLR transformation], and gene presence or relative abundance), including adjustment for potential confounders determined *a priori* including (body mass index (BMI, continuous), education level (≤high school, some college and college graduate and postgraduate), number of live-born children (never had a child, 1, 2, 3–4, ≥5), age at menopause (<40, 40–44, 45–49, 50–54, ≥55, still menstruating), first-degree female relatives with breast cancer (no, yes, unknown/missing) and consumption of alcohol from alcoholic drinks (g/day)). All variables were collected at baseline except for the age at sample collection since a questionnaire was not included at the time of sample collection. We standardized the alpha diversity and relative abundance measures by subtracting the overall mean in the controls and dividing by the standard deviation. We also calculated quartiles of alpha diversity based on the distribution in controls. The Microbiome Regression-Based Kernel Association Test (MiRKAT) [32] was used to evaluate the associations between the taxonomic beta diversity matrices and breast cancer status. To control for multiple comparisons in all exploratory analyses, we calculated q-values using the Benjamini-Hochberg procedure. We also evaluated the association between the presence or relative abundance of the periodontal pathogen complexes [33] and breast cancer. Specifically, we assessed the presence or cumulative relative abundance of any red (*Tannerella forsythia, Porphyromonas gingivalis* and *Treponema denticola)* and/or orange (*Prevotella intermedia, Prevotella nigrescens, Fusobacterium nucleatum, Fusobacterium periodonticum, Campylobacter gracilis, Campylobacter rectus, Campylobacter showae, Eubacterium nodatum* and *Streptococcus constellatus*) complex pathogens within individuals. To compare our results with our previous cross-sectional analysis using 16S rRNA gene sequencing [16], we additionally characterized taxonomy at the genus-level and compared our associations with the presence of 64 genera and relative abundance of 7 genera significantly associated with invasive breast cancer.

Additional supplementary analyses were conducted. First, we conducted a stratified analysis by time to breast cancer diagnosis (≤2 years or >2 years from sample collection). We selected this cutoff a priori as a pragmatic sensitivity analysis to evaluate possible reverse causation, rather than as a biologically established threshold. Among women diagnosed within 2 years, we further evaluated, at the species level, the presence of four genera that were significantly inversely associated with breast cancer in a previous report and replicated in our study. In addition, we excluded *in situ* breast cancer cases and their matched controls to evaluate alpha and beta diversity associations with invasive breast cancer. Finally, we restricted our analysis to estrogen receptor–positive (ER+) and progesterone receptor–positive (PR+) breast cancer cases and conducted conditional logistic regression models to evaluate associations between the alpha and beta diversity indices and the risk of breast cancer.

## Results

The characteristics of cases and controls are presented in Table 1. In general, cases and controls had similar demographic and other lifestyle characteristics including education level, BMI, alcohol consumption categories, number of live-born children and first-degree female relatives with breast cancer. Of the breast cancer cases, 256 (80.5%) and 206 (64.8%) had ER+ and PR+ tumors, respectively, while 251 (78.9%) of the cases were classified as invasive.

**Table 1.** Characteristics of women by breast cancer status, NIH-AARP diet and health study.

Variable	Incident cases		Matched controls	
	Mean/N	SD/(%)	Mean/N	SD/(%)
*Age at sample collection* ^a^				
<65 yrs	57	17.9	57	17.9
65–69 yrs	82	25.8	81	25.5
70–74 yrs	106	33.3	107	33.6
≥75 yrs	73	23.0	73	23.0
*Self-reported race & ethnicity* ^a^				
Non-Hispanic White	300	94.3	300	94.3
Other race and ethnicity groups^b^	18	5.7	18	5.7
*Education*				
<High school	63	19.8	75	23.6
High school and some college	118	37.1	115	36.2
College graduate and postgraduate	137	43.1	128	40.3
*BMI category*				
<25	145	45.6	148	46.5
25–29	106	33.3	103	32.4
≥30	67	21.1	67	21.1
*Number of live-born children*				
Never had a child	52	16.4	47	14.8
1	31	9.7	38	11.9
2	88	27.7	73	23.0
3–4	114	35.8	121	38.1
≥5	33	10.4	39	12.3
*Age at menopause*				
<40 yrs	45	14.2	54	17.0
40–44 yrs	39	12.3	46	14.5
45–49 yrs	61	19.2	60	18.9
50–54 yrs	125	39.3	124	39.0
≥55 yrs	30	9.4	17	5.3
Still menstruating	18	5.7	17	5.3
*First-degree female relatives with breast cancer*				
No	250	78.6	264	83.0
Yes	54	17.0	42	13.2
Unknown/missing	14	4.4	12	3.8
*Cigarette use* ^a^				
Never	134	42.1	134	42.1
Former	132	41.5	132	41.5
Current	38	11.9	38	11.9
Unknown/missing	14	4.4	14	4.4
*Alcohol from alcoholic drinks (beer, wine, liquor) (g/day)*				
0	57	17.9	70	22.0
>0–1	63	19.8	77	24.2
>1–2	58	18.2	53	16.7
>2–5	46	14.5	35	11.0
>5–10	32	10.1	22	6.9
>10	62	19.5	61	19.2
*Estrogen receptor status*				
Positive/elevated	256	80.5		
Negative/normal	30	9.4		
Borderline/missing	32	10.1		
*Progesterone receptor status*				
Positive/elevated	206	64.8		
Negative/normal	72	22.6		
Borderline/missing	40	12.5		
*Breast cancer behavior*				
*In situ*	67	21.1		
Invasive	251	78.9		

^a^ Matching factor.

^b^ The questionnaire included race and ethnicity category options of Asian/Pacific Islander/American Indian/Alaskan Native, Hispanic, non-Hispanic Black, non-Hispanic White, or missing/unknown.

### Alpha and beta diversity were generally not prospectively associated with breast cancer risk

No microbial community-level associations were observed with breast cancer incidence (MiRKAT *p* > 0.05) for either beta-diversity matrix (Bray–Curtis and Jaccard distances; Figure 1). Similarly, no associations were observed between observed species or the Shannon index with breast cancer in unadjusted (Figure 1) or adjusted (Table S1) analyses. However, a statistically significant inverse association was observed between Pielou's evenness and breast cancer risk. After adjustment for confounders, for each SD increase in Pielou's evenness, the odds of breast cancer decreased by 18% (95% CI = 0.70–0.95, *p* = 0.010). A similar trend was observed when Pielou's evenness was categorized into quartiles with an OR for breast cancer of 0.58 (95% CI = 0.35–0.96) for the highest quartile compared to the lowest quartile (Table S1).

**Figure 1.**
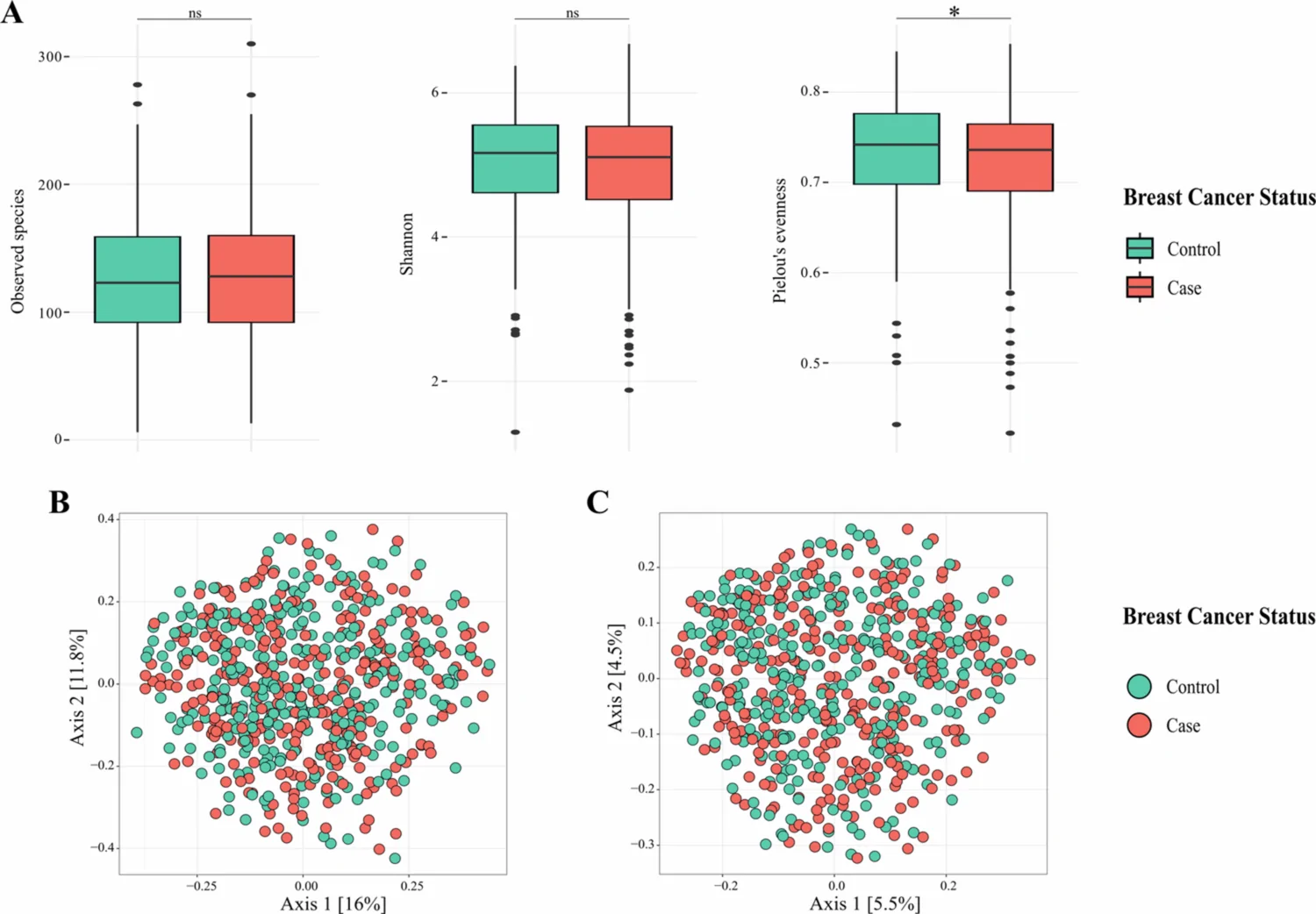
Comparison of microbial diversity metrics by breast cancer status. (A) Boxplot of the alpha diversity metrics by breast cancer status. (B) PCoA plot based on Bray–Curtis distance matrix. (C) PCoA plot based on Jaccard distance matrix. ns = not statistically significant. *Wilcoxon test significance *p* < 0.05.

### No genus-, species-, gene-, or pathway-level associations with overall breast cancer risk

Neither the presence nor the relative abundance of bacterial species clustered into periodontal disease complexes was significantly associated with breast cancer risk (Table S2). Similarly, after adjusting for multiple comparisons using a 5% false discovery rate (FDR) threshold, no statistically significant species-level associations with breast cancer were observed for presence, relative abundance, or CLR-transformed relative abundance. (Table S2). For functional analyses, no associations were found between the relative abundance or presence of included microbial genes or pathways and breast cancer risk (Table S3).

In our previous case-control study in Ghana, the presence of 64 genera was associated with breast cancer [16]. In the current prospective analysis, 23 out of the 64 genera were detected (Table S4). Similarly, of the seven genera previously associated with breast cancer based on relative abundance, six were detected in the present dataset. However, in this prospective analysis, neither the presence nor the relative abundance of these genera was associated with breast cancer risk (Table S4).

### Multiple oral microbiome associations with breast cancer for women diagnosed within 2 years of sample collection

A total of 97 women were diagnosed with breast cancer within two years of sample collection, with a mean time to diagnosis of 0.93 years. For every SD increase in Shannon index and Pielou's evenness, the odds of breast cancer decreased by 0.65 (95% CI = 0.44–0.98, *p* = 0.038) and 0.60 (95% CI = 0.41–0.89, *p* = 0.012), respectively. Moreover, breast cancer cases had statistically different microbial communities compared to controls based on the MiRKAT models (Bray–Curtis *p* = 0.0255; Jaccard *p* = 0.0209) (Table S5). However, exploratory analyses at the species level (Table S5), gene level and pathway level (data not shown) showed no significant associations either for the presence or the relative abundance of species, genes or pathways.

At the genus-level, the presence of 6 out of the 23 genera previously associated with the odds of breast cancer showed consistent inverse associations in our analysis; however, only three were statistically significant (e.g. *Treponema:* OR = 0.37; 95% CI = 0.17–0.78, *p =* 0.010; Figure 2). In contrast, the presence of *Catonella* exhibited an association in the opposite direction to that reported earlier (this study: OR = 2.19; 95% CI = 1.03–4.68, *p =* 0.042, previous study: OR = 0.42; 95% CI = 0.29–0.60, *p =* 0.0002; Figure 2).

**Figure 2.**
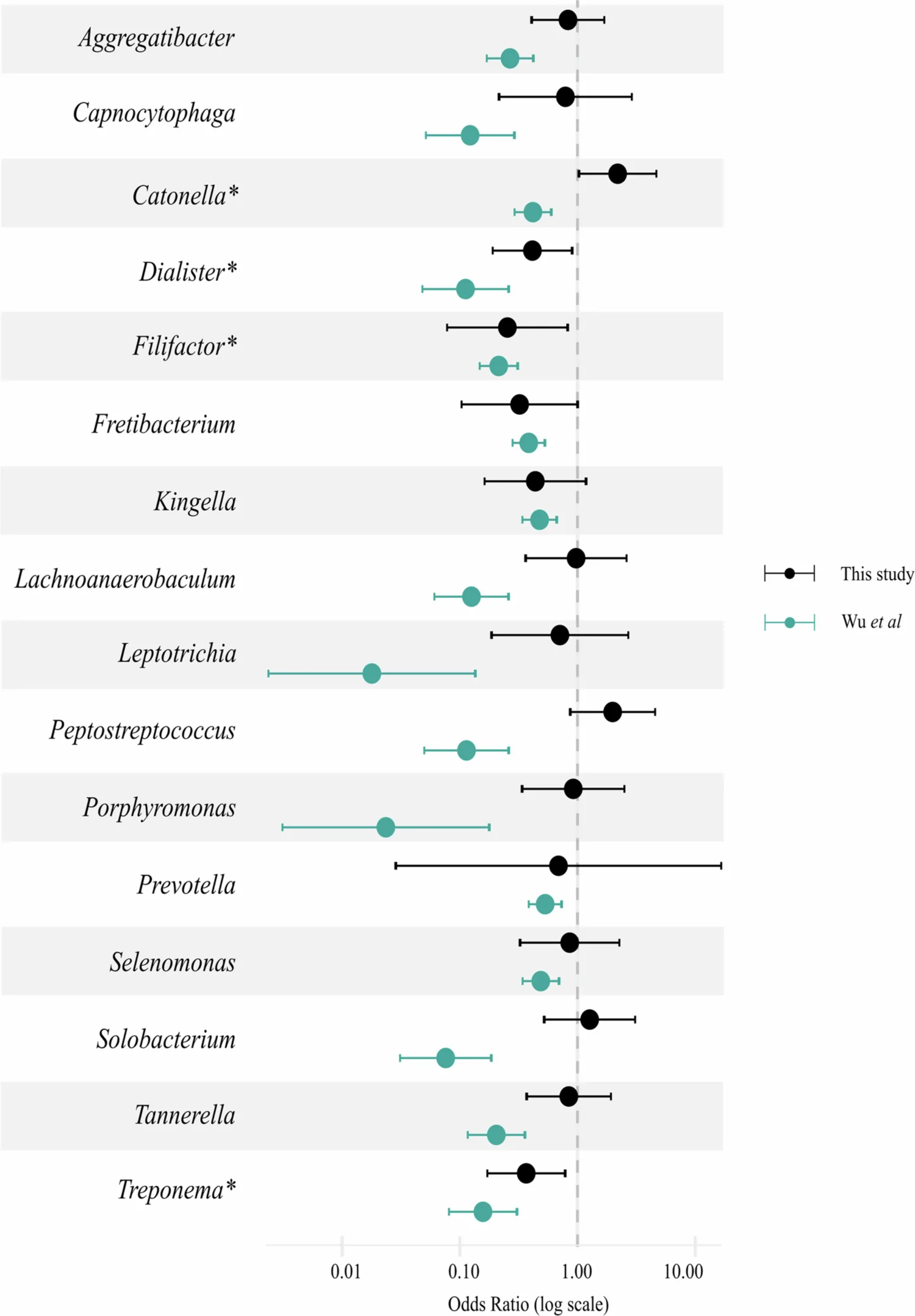
Forest plot showing associations between the presence of genera and breast cancer odds among women diagnosed within two years of sample collection. 23 genera previously reported to be associated with breast cancer [16] were evaluated in this study. Seven genera were excluded due to low prevalence, as they did not meet the inclusion criteria. Odds ratios and 95% confidence intervals from the current study are shown in black, while previously reported associations are displayed in gray for comparison. *Conditional logistic regression significance *p* < 0.05.

### No oral microbial associations with breast cancer in women diagnosed after 2 years from sample collection

Among the 221 women diagnosed with breast cancer more than two years after sample collection, no associations were observed between microbial diversity measures (alpha or beta) and breast cancer risk (Pielou's evenness: OR = 0.88, 95% CI = 0.72–1.06, *p* = 0.173; Table S5). Similarly, neither the presence nor the relative abundance of individual species (Table S5) or genes (data not shown) was associated with the risk of breast cancer. Finally, none of the genera previously reported to be linked to breast cancer showed an association in our analysis (data not shown).

### Few associations between microbial diversity and invasive, ER+, or PR+ breast cancer risk

When the analysis was restricted to invasive cases (*n* = 251) and their matched controls, inverse associations were identified with breast cancer risk for the Shannon index and Pielou's evenness (OR = 0.83, 95% CI = 0.69–0.99, *p* = 0.039; and OR = 0.80, 95% CI = 0.67–0.99, *p* = 0.014, respectively; Table S6). Notably, these associations were stronger when analyses were restricted to women diagnosed within two years of sample collection (*n* = 73) (Shannon: OR = 0.45, 95% CI = 0.25–0.82, *p* = 0.009; Pielou's evenness: OR = 0.42, 95% CI = 0.23–0.76, *p *= 0.005), but were not present in women diagnosed more than two years after sample collection (Table S6). Similar results were obtained for Pielou's evenness among ER+ cases (*n* = 256; OR = 0.83, 95% CI = 0.70–0.99, *p* = 0.040) and PR+ cases (*n* = 206; OR = 0.82, 95% CI = 0.67–0.99, *p* = 0.045), which likewise persisted only among women diagnosed within 2 years of sample collection (Table S6). In this subgroup, additional inverse associations were also observed between the Shannon index and the odds of breast cancer for both ER+ and PR+ cases (OR = 0.53, 95% CI = 0.31–0.91, *p* = 0.021; and OR = 0.35, 95% CI = 0.15–0.81, *p* = 0.014, respectively; Table S6). However, no associations were observed between the beta diversity and breast cancer risk in any group (Table S6). Additionally, neither the relative abundance nor the presence of any species was found to be associated with breast cancer in any subgroup (Table S6). Finally, none of the previously reported genera were associated with breast cancer in any subset (data not shown).

## Discussion

In this prospective, incidence density-matched case-control study nested within the NIH-AARP Diet and Health Study, we found that oral microbial metrics, including alpha and beta diversity, and genus-, species- and gene-level features, were generally not prospectively associated with postmenopausal breast cancer risk. However, an inverse association between one measure of alpha diversity, Pielou's evenness, was observed with breast cancer risk, but this association was mainly restricted to women diagnosed within two years of oral sample collection. In this subgroup of women diagnosed within two years of oral sample collection, several microbial measures, including alpha and beta diversity indices, were associated with breast risk, and previously reported genus-level associations from a Ghanaian case-control study were replicated (e.g. the presence of *Treponema, Filifactor* and *Dialister* were all associated with reduced breast cancer risk). No significant associations were observed among women diagnosed more than two years after sample collection. Similarly, the supplemental analyses restricted to invasive, ER+ and PR+ breast cancer cases indicated that any observed associations were largely driven by the subset of cases diagnosed within the first two years of follow-up.

Previous studies examining the relationship between the oral microbiome and breast cancer risk have primarily used case-control designs [16,18,19,34], which are susceptible to reverse causation. For instance, our recent case-control study conducted in Ghana, Africa, involving 369 breast cancer cases and 419 population-based controls, identified strong associations between multiple microbial metrics and breast cancer risk using 16S rRNA gene sequencing [16]. That study, conducted in a younger population (mean age 47.8), with approximately equal distribution of pre- and postmenopausal women, and with more than 50% of breast cancer cases having grade 3 tumors, demonstrated a strong inverse association between microbial alpha diversity and odds of breast cancer, along with notable differences in beta diversity metrics between cases and controls. A major limitation of this study was that biospecimens were collected after breast cancer diagnosis. Accordingly, the observed associations may reflect oral microbiome perturbations related to factors such as disease severity, rather than etiologically relevant pre-diagnostic differences. Although geographical, social and cultural factors may influence the oral microbiome [35,36], oral microbiome profiles are likely distinct between African and North American populations, and the oral collection methods were not the same between the two studies, we observed some similar associations among women diagnosed within two years of sample collection, who may have had or been in the early stages of breast cancer. These results suggest that previously reported associations may at least partly reflect reverse causation. In addition, oral health, periodontal status and hygiene may have contributed to the differences observed between cases and controls [37,38]; however, these factors were not collected in the present study and therefore could not be evaluated.

Furthermore, our findings are highly consistent with previous studies conducted in participants from North America, which showed no differences in the taxonomic composition of the oral microbiome between cases and controls [16,19,34]. For instance, Wang et al. evaluated oral rinse samples from 55 breast cancer cases and 21 non-cancer controls, the majority of whom were white (74%) and postmenopausal (59%). The study found no significant differences in alpha diversity, overall community structure, or the relative abundance of any genus between cancer and non-cancer patients [34]. Nearing et al. analyzed samples from two predominantly white Canadian cohorts (~90%) (the Atlantic Partnership for Tomorrow's Health (PATH) and Alberta's Tomorrow Project (ATP) cohorts). The study included both retrospective (54 breast cancer cases and 218 controls) and prospective samples (136 incident breast cancer cases and 136 healthy controls), finding no significant differences in oral microbiome diversity or composition. The authors concluded that the oral microbiome is unlikely to serve as an effective population-based risk marker for breast cancer [18]. Finally, McCune et al included samples from 73 breast cancer cases, 32 ductal carcinoma *in situ* cases (DCIS) and 49 healthy controls from the US, showing that the oral microbiota is very similar between healthy women and women with DCIS or invasive breast cancer [18].

Reverse causation remains a plausible explanation for the inverse associations observed in this study (women diagnosed within 2 years of sample collection) and in our previous study in Ghana. Because neither study included post-treatment oral samples, any breast cancer treatment would not explain our findings. Nevertheless, disease-related changes in the oral microbiome are biologically plausible. Possible contributors in the context of breast cancer include systemic inflammation, immune perturbation and alterations in the local oral environment [39]. Evidence that circulating estrogens and estrogen metabolites are associated with both the fecal and oral microbiome in postmenopausal women [40] supports the possibility that host hormonal factors, including unmeasured menopausal hormone therapy use, may contribute to microbial variation and residual confounding. Taken together, these observations support the plausibility that host- or disease-related factors associated with occult or developing breast cancer could contribute to differences in the oral microbiome, including periodontal pathogens. Therefore, the inverse associations observed between some periodontal pathogens and breast cancer among women diagnosed within 2 years of sample collection should be interpreted with caution, as they may reflect microbial alterations related to occult or developing disease.

Although the oral microbiome may contribute to carcinogenesis through inflammation, immune dysregulation and altered cell signaling, the importance of these mechanisms may vary across cancer sites [41,42]. In postmenopausal women, breast cancer is also strongly influenced by age-related exposures and other metabolic and hormonal factors [2,3], which may make any modest oral-microbiome signal difficult to detect. It is also possible that a single oral sample collected later in life may not capture the relevant etiologic window for breast carcinogenesis [19]. This may help explain why evidence supporting the oral microbiome as a biomarker for breast cancer remains limited. In addition, the isolated inverse association observed for Pielou's evenness, in the absence of associations for observed richness, Shannon diversity, beta diversity, or individual microbial features after multiple-comparison correction, may reflect a subtle shift in the balance of the oral community rather than a difference in taxonomic richness or a strong signal driven by specific taxa. This finding should therefore be interpreted with caution.

This study has multiple strengths, including the use of shotgun metagenomic sequencing, which enabled analyses at both the species and gene levels. Additionally, the robust study design included prediagnostic biospecimens and a rigorous matching process that accounted for key breast cancer risk factors, along with statistical models that adjusted for multiple potential confounders. However, some limitations should be acknowledged. First, we did not collect information about the oral health status and oral hygiene practices, so we cannot exclude the possibility of residual confounding. Second, this study only included postmenopausal breast cancer cases, which tend to be hormone receptor positive; given the distinct etiology of pre- and postmenopausal breast cancer [43], we cannot extend these results to pre-menopausal breast cancer risk. It is worth noting that menopausal status was assessed only at baseline (1995–1996), and updated menopausal status at oral sample collection in 2005 was unavailable, although the 18 breast cancer cases who reported being pre-menopausal at baseline were 60.0 to 71.7 years old at sample collection and were therefore likely postmenopausal at that time, some misclassification remains possible. Third, although the NIH-AARP Diet and Health study cohort offers a broad geographic representation across different states and cities in the US, it is not racially or ethnically diverse, limiting its generalizability to other populations. Fourth, 34 women were excluded from the main analysis due to DNA extraction issues or insufficient read counts, which may have reduced the study's statistical power. Given the minimum detectable ORs based on our current sample size, we cannot exclude the possibility that we were underpowered to detect modest associations with overall breast cancer risk. Finally, two blanks indicated some contamination; however, exclusion of these batches did not influence the results.

## Conclusions

Our findings suggest that the oral microbiome is not prospectively associated with breast cancer risk in postmenopausal women. Moreover, our results provide evidence that previously reported associations may be due to reverse causation. Future studies may wish to evaluate the association between the oral microbiome and breast cancer in pre-menopausal women.

## Supplementary Material

Fig_S2.tif Fig_S2.tif

Fig S1.tif Fig S1.tif

Supplementary material Supplementary figure captions.docx

Supplementary tables.xlsx Supplementary tables.xlsx

## Data Availability

The data underlying this article are available in the Sequence Read Archive (SRA) under the BioProject ID PRJNA1366739.
