# Supplementary material for: Oral microbiome and postmenopausal breast cancer risk: findings from the NIH-AARP diet and health study
Source: J Oral Microbiol. 2026 Oct 9;18(1):2728745. doi: 10.1080/20002297.2026.2728745 (PMC13656656; doi:10.1080/20002297.2026.2728745)
Supplement: Supplementary file 1 [file ZJOM_A_2728745_SM6533.docx]

**Figure S1.** Flow diagram of the sample selection process. The figure illustrates the number of samples excluded at each step of the analysis. *One participant contributed a sample that was included both as a case and as a control.

**Figure S2.** (A) Rarefaction curves showing observed species richness, Shannon diversity, and Pielou’s evenness. (B) Principal coordinates analysis (PCoA) plots of samples by sample type based on Bray–Curtis (left) and Jaccard (right) distance matrices. Paired replicates cluster closely, indicating high intra-batch reliability
